# Rare Causes of Cerebral Venus Sinus Thrombosis: A Systematic Review

**DOI:** 10.3390/life13051178

**Published:** 2023-05-12

**Authors:** Rafaella Theologou, Antonios Nteveros, Artemios Artemiadis, Konstantinos Faropoulos

**Affiliations:** 1 Department of Neurology, Nicosia General Hospital, 2029 Nicosia, Cyprus; 2 Medical School, University of Cyprus, 1678 Nicosia, Cyprus; 3 Department of Neurosurgery, Nicosia General Hospital, 2029 Nicosia, Cyprus

**Keywords:** cerebral venus sinus thrombosis, rare cause, idiopathic, inflammatory, systematic review

## Abstract

**Background:** Cerebral venous sinus thrombosis (CVST) is a rare manifestation of thrombosis commonly caused by thrombophilia, hormonal-related factors, non-cerebral malignancy, and hematologic diseases. The aim of this review was to identify and summarize rare CVST cases. **Methods:** A literature search of the Medline database was performed in November 2022. CVST cases of a common cause were excluded. Demographic and clinical data were extracted. Eligible cases were categorized into inflammatory, primary CNS tumors, post-operative/traumatic, and idiopathic groups to allow statistical group comparisons. **Results:** 76 cases were analyzed. Idiopathic CVST was most frequently reported followed by inflammatory, post-traumatic/operative and primary CNS tumor causes. The intracranial hemorrhage rate was 23.7% and it was found to increase in the inflammatory group (45.8%). Anticoagulation was used in the majority of cases and it was significantly related to better outcomes. A low rate of anticoagulation use (43.8%) was found among CVST cases in the post-operative/traumatic group. The overall mortality rate was 9.8%. 82.4% of patients showed significant early improvement. **Conclusions:** Most rare CVST cases were either of idiopathic or inflammatory origin. Interestingly, hemorrhage occurred often he idiopathic CVST cases. A low rate of anticoagulation use in neurosurgical CVST cases after trauma or head surgery was observed.

## 1. Introduction

Cerebral venous sinus thrombosis (CVST) is a rare manifestation of thrombosis involving brain dural venous sinuses and/or cerebral veins, mainly affecting young females [1]. The incidence of CVST varies among studies and between different ethnic groups. In adults, the annual incidence of CVST is 2–5 cases per million, albeit the number of cases is increased in developing countries most likely due to the high incidence of infectious diseases [1].

In the clinical settings, CVST patients present with symptoms of headache (88.8%), seizures (39.3%), paresis (37.2%), papilledema (28.3%), and mental status changes (22%) [2]. The unspecific nature of these symptoms, especially when the headache is the sole manifestation, renders diagnosis cumbersome and delays appropriate treatment for a median of 7 days [2]. The superior sagittal and transverse sinuses are most frequently found affected, followed by the internal jugular and cortical veins, while in over 60% of CVST cases more than one sinus is found thrombosed during vascular imaging [1]. Enhanced CT scan is the mainstay of CVST diagnosis with a sensitivity of 99% [3]. However, the increasing accessibility to MRI imaging has substituted CT scans for the diagnosis.

Therapeutic doses of low molecular weight heparin (LMWH) are the mainstay of CVST treatment even in the presence of CVST-related intracranial hemorrhage [4]. Thrombolysis and endovascular treatment have also been tested in small or case series studies, but they have shown ambiguous effects and have raised many safety concerns, especially for intracranial hemorrhage [5,6]. With regards to prognosis, about 5% of CVST cases die from complications mostly related to comorbidities and CVST-related intracranial hemorrhage or [2]. However, more than 90% of the survivors achieve relative independence in a median of 16 months after the event [2]. 

Inherited thrombophilia, hormonal-related factors (e.g., oral contraceptives, pregnancy, puerperium), non-cerebral malignancy, head and neck infections, and systemic and hematologic diseases have been recognized as common risk factors for CVST [1]. In 85% of cases, at least one risk factor is identified, otherwise, the CVST is considered idiopathic [7]. Oral contraceptive use is by far the most common risk factor, reported in more than 80% of women in various series and associated with a pooled estimate of approximately 6-fold increased risk of CVST [8]. Pregnancy or the puerperium is responsible for 5–20% of CVST, with an incidence of 12 cases per 100,000 deliveries [9,10]. Among inherited thrombophilia abnormalities related to CVST, the most prevalent are heterozygous FV Leiden and prothrombin G20210A polymorphisms [11]. Malignanca y, mainly non-cerebral solid tumor or hematological cancer, is also a risk factor in 7% of patients with CVST [12]. Autoimmune diseases, viral infections, primary central nervous system (CNS) tumors and neurosurgical procedures are very rare risk factofor of CVST [1,13,14,15,16].

To our knowledge and clinical experience, the rare causes or risk factors of CVST are often overlooked by clinicians. Also, the literature on the rare causes is scarce or parsimonious, especially regarding reviews or meta-analyses. In this systematic review, we aimed to focus on CVST cases of rare or idiopathic etiology only. To this aim, cases were grouped into four categories; inflammatory, primary central nervous system (CNS) tumor, post-operative or post-traumatic and idiopathic. The categories of the causes of CVST were formed based on the presumed etiopathogenesis. For example, the inflammatory category included rare diseases such as viral infections or autoimmune diseases (e.g., Behcet’s disease, ulcerative colitis or other systemic autoimmune diseases) that most likely induce thrombosis through the activation of the immune response. On the other hand, CNS tumors most likely cause CVST through mechanical pressure or cell infiltration in cerebral veins. The same mechanic phenomena may also explain CVST related to previous head operations or trauma. By grouping these rare cases of CVST further analysis was facilitated. As such, in this review a descriptive analysis along with group comparisons was presented further elucidating the matter of rare causes of CVST.

## 2. Materials and Methods

A literature search was performed on 11 November 2022 in the Medline database to identify cases of CVST. The search terms were “cerebral venous sinus thrombosis [Title/Abstract]” NOT “COVID-19 [Title/Abstract]”. Filters were applied: case reports, English, adult: 19+ years, humans. Publications describing CVST cases with common risk factors i.e., acquired (such as antiphospholipid syndrome) or inherited thrombophilia, hormonal-related factors (e.g., oral contraceptives, pregnancy, puerperium), localized or bacterial infections, hematologic diseases and systemic malignancies were excluded. Cases of CVST caused by drugs due to isolated reporting or unknown or doubtful mechanisms of drug-related CVST. Also CVST cases due to intracranial hypotension, mainly after lumbar puncture, were also excluded due to the well-known, albeit rare, complication of lumbar puncture Also, cases that were not retrieved as full texts were excluded. Case reports failing to report adequate work-up (i.e., thrombophilia, hormonal-related factors, and autoimmunity or infection screening tests) for CVST were also excluded. The remaining cases were grouped into the following categories: active inflammatory (autoimmune or viral infections), primary CNS tumors, post-operative/post-traumatic, and idiopathic after a consensus meeting of the authors. 

Extracted data included the following: gender, age (in years), location of thrombosis, the occurrence of ischemic infarct (yes/no) or intracranial hemorrhage (yes/no), CVST-related risk factor, use of anticoagulation (yes/no), other CVST-related treatment (i.e., thrombolysis, endovascular treatment), early (i.e., hospitalization period) and follow-up outcome (severely disabled/stable/improved/death/normal i.e., no neurological symptoms or signs). 

The demographic and clinical characteristics of the included cases were summarized by using descriptive statistics. Group comparisons were performed by using simple univariate non-parametric tests i.e., Mann–Whitney test, Kruskal–Wallis H test and Fisher’s exact test. The level of significance was set at 0.05. The analysis was performed with IBM SPSS Statistics for Windows, version 21.0. (IBM Corp, Armonk, NY, USA).

## 3. Results

The PRISMA flow diagram was constructed. A total of 320 registers were retrieved from MEDLINE. Five records were removed as duplicate, 13 could not be retrieved as full texts and 35 were excluded as non-original. In total, 262 records were screened. Twenty-seven were excluded. In these records, 292 cases were sought for retrieval. Among them, 216 were excluded. Forty-four cases had a common cause for CVST, mainly inherited or acquired thrombophilia. Twenty-three cases pertained to hormonal-related factors. Ten cases had a localized or bacterial infection. In 9 cases the cause of CVST was a hematologic disease, 6 cases had a systematic malignancy, 12 cases had confirmed intracranial hypotension, and in the vast majority no cause was reported. In the latter, publications failed to provide adequate information on the work-up tests. Thus, it was not evident if these CVST cases were of idiopathic origin if there was some other cause or risk factor. As such, a total of 63 publications describing 76 cases were analyzed since they met inclusion and exclusion criteria [17,18,19,20,21,22,23,24,25,26,27,28,29,30,31,32,33,34,35,36,37,38,39,40,41,42,43,44,45,46,47,48,49,50,51,52,53,54,55,56,57,58,59,60,61,62,63,64,65,66,67,68,69,70,71,72,73,74,75,76,77,78,79]. Table 1 summarizes the included cases. 

Among the 76 cases, 30 (39.5%) had an inflammatory cause of CVST, 16 (21.1%) patients suffered CVST after surgery or head trauma, 6 (7.9%) had a primary CNS tumor and 24 (31.6%) were identified as idiopathic CVST cases. Amongst the inflammatory cases, most patients had ulcerative colitis or neuro-Behcet’s disease and there were some isolated cases of autoimmune thyroiditis, idiopathic hypertrophic pachymeningitis, Sjogren’s disease, IgM nephropathy, and viral CNS infections, especially herpetic. In the neurosurgical category, half of the cases (i.e., eight) were found with CVST after head trauma and the other half had neurosurgical surgery before the thrombotic event.

In total, there were more males than females (50.2% vs. 40.8%, respectively). The mean age was 37.2 ± 15.1 years old (range: 18–77) and did not differ by gender (*p* = 0.876). Most cases (57/76, 75%) had thrombosis in two or more sinuses or cerebral veins. Twelve (15.8%) patients had CVST-related infarct and 18 (23.7%) suffered CVST-related intracranial hemorrhage. Gender did not significantly affect the possibility of CVST-related infarct (*p* = 0.34) or hemorrhage (*p* = 0.586), and neither was age (i.e., for infarct *p* = 0.874, for hemorrhage *p* = 0.327). Anticoagulation as acute treatment was used in 59 (78.7%) cases, while thrombolysis or endovascular treatment was performed in 12 cases (15.8%). With regards to early outcomes, 25 case reports failed to provide adequate information on the clinical status of the patients. In the remaining 51 cases, there were 5 deaths (9.8%) and 42 cases (82.4%) were significantly improved. Four patients (7.8%) showed clinical stability. The use of anticoagulation was significantly associated with more improvement than no anticoagulation (89.5% vs. 61.5%, *p* = 0.036). Although, all patients (100%) that received thrombolysis or endovascular treatment were found to improve compared to 78% among those patients without such therapy, the difference was not found significant (*p* = 0.176). With regards to long-term monitoring, the mean clinical follow-up time was 6.6 ± 6.4 months (range: 0.75–30 months) and clinical descriptions were available for only 34 cases. Most cases (16/34, 47.1%) were completely normal, 12/34 (35.3%) were clinically stable, 5/34 (14.7%) showed improvement and there was only 1 (2.9%) death after follow-up.

Table 2 summarizes the main characteristics of the included cases by category. The primary CNS tumor group was excluded from group comparisons due to its very small sample size. The age and gender did not differ between the three remaining groups (*p* = 0.244 and *p* = 0.675, respectively). CVST-related infarct occurrence was similar between the three groups (*p* = 0.788). However, intracranial hemorrhage was more frequent among patients with idiopathic CVST compared to those with inflammatory or post-traumatic/post-operative CVST (45.8% vs. 10% or 18.8%, respectively, *p* = 0.008). Anticoagulation treatment was conspicuously withheld in patients in the post-traumatic/post-operative group (only 43.8% of patients received treatment) compared to the inflammatory (82.8%) or idiopathic group (91.7%) (*p* = 0.001). However, this was not found to be translated into a worse early outcome (*p* = 0.52) compared to the other group, as 70% of neurosurgical cases were improved compared to 85.7% and 85% in the inflammatory and idiopathic groups respectively. Despite that, we must note that 30% of patients in this group died (compared to only 4.8% and 5% in the inflammatory and idiopathic groups, respectively). Finally, most cases (>50%) in all groups were normal or improved after follow-up (analyses was not performed due to the small number of cases and different follow-up time).

## 4. Discussion

This systematic review aimed to identify and summarize CVST cases of rare causes and to draw clinically useful conclusions by grouping these cases according to their presumed pathogenesis. Four groups were created: inflammatory, post-operative/traumatic, primary CNS tumors, and idiopathic. The main findings of this study are discussed below.

Among cases, idiopathic CVST was the most frequent followed by inflammatory, post-traumatic/operative, and primary CNS tumor. This was in accordance with previous research wherein idiopathic CVST accounts for 15% of all CVST cases [7]. However, in the present research, these differences may be misleading if we do not take into account that categories such as “inflammatory” are more inclusive than others like primary CNS tumor. In this context, this may also suggest that the “idiopathic” category most probably includes a variety of factors or thrombotic diseases, either misdiagnosed or unknown. 

Most cases in the inflammatory category had ulcerative colitis or neuro-Behcet’s disease or viral infections mostly varicella zoster virus (VZV). Inflammatory bowel disease and especially ulcerative colitis is a well-known prothrombotic disease, although CVST is a rare extraintestinal complication in these patients [13]. On the other hand, CVST is reported in 10–30% of neuro-Behcet’s disease cases [14]. Viral CNS infections and especially VZV rarely cause CVST but the incidence is to our knowledge unknown. Notably, in COVID-19 infection the frequency of CVST was about 3.5%. Inflammatory vasculopathy and the generation of prothrombotic factors are assumed to be the main culprit for the thrombotic events in inflammatory diseases [13,14,15]. 

CVST-related intracranial hemorrhage was found to occur in 23.7% of cases which is slightly less than the expected 30–40% for all CVST cases [1]. However, intracranial hemorrhage was more frequent among patients with idiopathic CVST (45.8%) compared to those with inflammatory (10%) or post-traumatic/post-operative (18.8%) CVST. Intracranial hemorrhage in CVST is thought to be caused by the rupture of venules due to high intravenous pressure. To our opinion, the higher incidence of hemorrhage in idiopathic CVST cases may be indicative of an underlying vasculopathy like that encountered in inflammatory diseases wherein inflammation causes disruption of the blood-brain barrier, oxidative stress, and endothelial dysfunction.

As expected, anticoagulation was the first-line treatment in our CVST cases, and it was significantly associated with better early outcomes. However, among patients in the post-traumatic/post-operative group, only 43.8% of patients received anticoagulation. Also, most CVST cases with Neuro–Behcet’s disease did not receive anticoagulation since, although still debatable, immunosuppression that suppresses venous inflammation is highly recommended in these patients. This most probably reflects the hesitancy of neurosurgeons to administer anticoagulation in the face of a possible hemorrhagic complication after surgery or a head trauma. Although anticoagulation is highly recommended even in CVST cases with brain hemorrhage, we think that, in the absence of pertinent studies, the decision would be taken on an individual basis according to the neurosurgical patients’ comorbidities [4]. In our study, only 12 patients received thrombectomy or endovascular treatment thus no safe conclusions can be made.

Finally, with regard to prognosis, 9.8% of patients died which is similar to the expected rate of fatality [1,2]. Despite that, we must note that 30% of patients in the neurosurgical group died, which could be explained by the severity of these cases due to co-morbidities. Also, the rate of both early and late improvement was high, but we must note that most case reports failed to objectify the clinical status by using scales such as the modified Rankin scale or others. As such, we could not draw safe conclusions regarding prognosis and the presented analyses should not be overstated.

This study suffers from several limitations. Firstly, there is always the chance that rare CVST cases have been missed although our search strategy and the eligibility criteria were not strict. Moreover, under-reporting issues or grey literature might also hinder case inclusion. Secondly, the grouping of cases was performed according to the assumed pathogenesis to allow comparisons and conclusions. However, this strategy produced uneven and heterogenous groups thus, no safe conclusions for group or individual disease occurrence can be made. Thirdly, 112 cases were excluded from the analysis, since no clear cause or risk factor was reported or was inadequately explored. It is very likely, that these cases were true idiopathic CVST cases and thus missed by this study. Finally, comparative statistical analysis was performed in a small number of cases thus there is always the chance of type I or II error.

In a nutshell, most rare CVST cases were either of idiopathic or inflammatory origin. An interesting finding of this study was that idiopathic CVST cases were more likely to suffer intracranial hemorrhage, which may be indicative of an underlying misdiagnosed or unknown inflammatory process as discussed above. Anticoagulation remained the mainstay of rare CVST cases and was related to better outcomes. However, we found a low rate of anticoagulation use in neurosurgical CVST cases after trauma or head surgery.

## Figures and Tables

**Table 1:** Summary of case characteristics.

Reference	Age	Gender	Anatomical Location	Infarct	Haemorrhage	Cause	Anticoagulation Treatment	Thrombolysis or Endovascular Treatment	Early Outcome	Follow-Up Outcome
[17]	31	male	superior sagittal sinus, straight sinus, and both transverse sinuses	Yes	No	Grave’s disease	Yes	No	NA	18 months, normal
[18]	27	male	left sagittal sinus	Yes	No	Ulcerative colitis	Yes	No	improved	NA
[19]	27	female	left transverse, sigmoid sinus	No	No	Ulcerative colitis	Yes	No	improved	6 months, normal
[19]	45	male	left temporal venous, left transverse, sigmoid sinus	No	No	Ulcerative colitis	Yes	No	NA	6 months, normal
[20]	52	female	superior sagittal, straight, left transverse sinus	No	No	Idiopathic hypertrophic pachymeningitis	Yes	Yes	improved	1 year, normal
[21]	23	male	right transverse sinus, sagittal sinus	No	No	IgM nephropathy	Yes	No	NA	1 year, normal
[22]	50	female	left transverse sinus	No	No	Sjögren’s syndrome	Yes	No	NA	6 months, normal
[23]	75	male	left sigmoid and transverse sinus	Yes	No	Acute herpes zoster	Yes	No	improved	NAs
[24]	32	female	right transverse, sigmoid sinus, and straight sinus, superior sagittal sinus, left transverse sinus	No	No	Grave’s disease	Yes	Yes	improved	3 months, improved
[15]	20	male	sagittal sinus, straight sinus, bilateral frontal parasagittal cortical veins, transverse and sigmoid sinuses, and left internal jugular vein,	No	No	Varicella zoster virus	Yes	No	improved	2 months, normal
[25]	25	male	right transverse sinus	No	No	Ulcerative colitis	Yes	No	improved	5 months, normal
[26]	20	male	widespread	No	No	Varicella zoster virus	Yes	No	NA	NA
[27]	28	female	superior sagittal sinus, sigmoid sinus, and internal jugular vein	No	No	Neuro–Bechet’s disease	No	No	improved	NA
[28]	44	female	sagittal, right transverse, and sigmoid sinuses	No	No	HIV	Yes	No	improved	NA
[29]	22	male	right sigmoid sinus	Yes	Yes	Neuro-Bechet’s disease	No	No	improved	NA
[30]	19	female	right transverse, right sigmoid, left transverse and straight sinuses	No	Yes	Japanese encephalitis	Yes	No	improved	NA
[31]	28	male	left sigmoid sinus	No	No	Hyperthyroidism, chronic thyroiditis	Yes	No	stable	NA
[32]	55	female	right internal jugular vein, right sigmoid, transverse, superior sagittal sinus	No	No	Invasive fibrous thyroiditis	Yes	No	improved	3 months, NA
[33]	29	male	frontoparietal region	No	No	ANCA and anti-GBM positivity	Yes	No	stable	3 months, NA
[34]	37	male	both sigmoid sinuses	No	No	Neuro-Bechet’s disease	No	No	improved	9 months, NA
[35]	38	female	Left sigmoid, transverse	No	No	Neuro-Bechet’s disease	No	No	death	death
[36]	27	male	Left transverse sinus	No	No	JC Polyomavirus	Yes	No	improved	9 months, NA
[37]	26	female	bilateral transverse sinus, left venous sinus thrombosis	Yes	No	Neuro-Bechet’s disease	Yes	No	NA	NA
[38]	31	male	superior sagittal sinus	No	No	HSV encephalitis	Yes	No	improved	1 year, normal
[39]	30	male	superior sagittal sinus	No	No	Neuro-Bechet’s disease	Yes	No	NA	NA
[40]	18	male	superior sagittal sinus, both transverse sinuses	No	No	Neuro-Bechet’s disease	No	No	improved	1 year, normal
[40]	26	female	left sigmoid sinus	No	No	Neuro-Bechet’s disease	NA	No	NA	NA
[41]	41	male	superior sagittal sinus and adjacent cortical veins	No	No	Hashimoto’s thyroiditis	Yes	No	NA	2 months/stable
[42]	45	male	right transverse sinus	No	Yes	Autoimmune hyperthyroidsm	Yes	No	improved	1 year, stable
[43]	41	female	right transverse sinus, sigmoid sinus, and jugular vein	No	No	Pachymeningitis	Yes	No	improved	1 month, stable
[44]	21	female	left transverse, sigmoid, jugular bulb	No	No	Glioblastoma	Yes	No	NA	NA
[44]	61	male	left transverse, sigmoid, left internal jugular vein	No	No	Glioblastoma	Yes	No	NA	12 months, NA
[44]	32	male	bilateral transverse, sigmoid	No	No	Pilocytic astrocytoma	Yes	No	NA	NA
[44]	75	male	left sigmoid	No	No	Atypical meningioma	Yes	No	NA	NA
[44]	60	male	right transverse, sigmoid, internal jugular vein	No	No	PCNSL	Yes	No	NA	9 months, death
[44]	21	male	superior sagittal sinus, right frontal	No	Yes	Oligodendroglioma	Yes	No	NA	3 months, stable
[45]	44	male	Left lateral sinus, right proximal lateral sinus	No	No	Post glomus jugulare tumor surgery	Yes	No	NA	NA
[46]	20	male	sigmoid sinus, jugular bulb thrombosis	No	Yes	Post-traumatic	No	Yes	improved	6 months, stable
[47]	43	female	sagittal sinus, right transverse sinus, sigmoid sinus, right internal jugular	No	No	Complications after transsphenoidal surgery	Yes	No	improved	NA
[48]	42	male	right transverse sinus	No	No	Post-traumatic	No	No	NA	3 months, NA
[49]	37	female	right sigmoid, transverse, and straight sinuses and the posterior part of the superior sagittal sinus thrombosis	No	No	Post-traumatic	No	No	death	NA
[50]	29	male	multible cerebral venus thrombosis	Yes	Yes	Post-traumatic	Yes	No	NA	NA
[51]	45	female	superior sagittal sinus, bilateral transverse, and sigmoid sinus	No	Yes	Post-operative	No	No	death	NA
[52]	21	female	right jugular vein, sigmoid, and transverse sinuses, superior sagittal sinus	No	No	Post-operatively after right trans-labyrinthine craniotomy	Yes	Yes	improved	6 weeks, stable
[53]	NA	male	superior sagittal sinus	No	No	Post-traumatic	No	No	improved	NA
[54]	38	male	superior sagittal	No	No	Post-traumatic	No	No	NA	NA
[55]	30	male	sigmoid and transverse sinuses suggestive of right IJV thrombosis	No	No	Therapeutic ligation of the internal jugular vein during neck dissection	Yes	No	improved	1 year, normal
[56]	25	female	superior sagittal sinus, right proximal transverse sinus	No	No	Post-traumatic	No	No	improved	5 months, normal
[57]	55	male	superior sagittal and straight sinuses	Yes	No	Craniotomy	No	No	death	NA
[57]	21	male	superior sagittal and straight sinuses	No	No	Post-traumatic	No	Yes	NA	NA
[58]	67	male	superior sagittal sinus, transverse sinus	No	No	Post-operative	Yes	No	NA	NA
[59]	28	female	right lateral and the superior sagittal sinuses	No	No	Post-operative	Yes	No	improved	3 months, improved
[60]	38	female	left transverse-sigmoid sinus extending into the left internal jugular vein	No	No	Idiopathic	Yes	No	NA	1 year, NA
[61]	77	male	superior sagittal sinus and right transverse sinus	No	No	Idiopathic	Yes	No	death	NA
[62]	72	male	right transverse and sigmoid sinus, left a lateral mass of the C1 vertebra	No	No	Idiopathic	Yes	No	improved	NA
[63]	32	male	right jugular and transverse venous sinus	No	No	Idiopathic	Yes	No	improved	6 months, NA
[64]	45	female	right sigmoid and transverse sinuses	No	No	Idiopathic	Yes	No	stable	Weeks, normal
[65]	57	female	sagittal sinus	No	Yes	Idiopathic	No	No	stable	18 months, some disability
[66]	30	female	superior sagittal sinus, left transverse sinus	Yes	No	Idiopathic	Yes	No	improved	2,5 years, stable
[67]	44	female	left transverse sinus, internal jugular vein,	No	No	Idiopathic	Yes	No	improved	1 month, normal
[68]	47	female	sphenoparietal sinus and superficial middle cerebral vein	No	Yes	Idiopathic	No	No	improved	6 months, some disability
[69]	35	male	left cavernous sinus	No	No	Idiopathic	Yes	No	improved	NA
[70]	24	male	sagittal sinus, the vein of Galen, straight sinus, left transverse sinus, and left sigmoid sinus	yes	Yes	Idiopathic	Yes	No	improved	2 Months, stable
[71]	21	female	posterior part of superior sagittal sinus, left transverse sinus	No	No	Idiopathic	Yes	No	NA	25 Days, NA
[72]	31	female	transverse sinuses, superior sagittal sinus, right internal jugular vein	No	No	Idiopathic	Yes	No	NA	NA
[73]	46	male	superior sagittal, draining veins, transverse, and sigmoid sinuses	Yes	Yes	Idiopathic	Yes	No	improved	6 months, stable
[73]	35	male	superior sagittal sinus	Yes	Yes	Idiopathic	Yes	No	improved	4 months, stable
[74]	25	male	superior sagittal sinus, right transverse sinus, sigmoid sinus, internal jugular vein	No	Yes	Idiopathic	Yes	Yes	improved	3 months, stable
[74]	22	female	superior sagittal, left transverse, left sigmoid, straight sinuses	No	Yes	Idiopathic	Yes	Yes	improved	3 weeks, normal
[75]	46	male	superior sagittal sinus, cortical veins, right lateral and sigmoid sinuses, transverse, internal jugular vein	No	Yes	Idiopathic	Yes	Yes	improved	2 months, some disability
[76]	39	female	straight sinus, left transverse-sigmoid and superior sagittal sinus	No	Yes	Idiopathic	Yes	No	improved	1 month, normal
[77]	21	female	Left venous sinuses, superior sagittal	No	No	Idiopathic	Yes	Yes	improved	30 days, some disability
[26]	54	female	superior sagittal, straight, right transverse, sigmoid sinuses	Yes	No	Idiopathic	Yes	Yes	improved	NA
[78]	26	male	straight sinus, superior sagittal sinus, torcula, transverse sinus, internal cerebral veins, a vein of Galen, vein of Rosenthal	No	Yes	Idiopathic	Yes	Yes	improved	NA
[78]	32	male	superior sagittal sinus	No	Yes	Idiopathic	Yes	Yes	NA	NA
[79]	67	male	straight sinus	No	No	Idiopathic	Yes	No	improved	NA

**Table 2:** Main characteristics of the included cases by risk factor categories.

	Inflammatory	Primary CNS Tumor	Post-Operative or Traumatic	Idiopathic
N, (%)	30 (39.5)	6 (7.9)	16 (21.1)	24 (31.6)
Mean age ± SD (range)	33.7 ± 12.9 (18–75)	45 ± 23.3 (21–75)	36 ± 13.4 (20–67)	40.3 ± 16 (21–77)
Females N, (%)	12 (40)	1 (16.7)	6 (37.5)	12 (50)
CVST-related Infarct N, (%)	5 (16.7)	0 (0)	2 (12.5)	5 (20.8)
CVST-related Hemorrhage N, (%)	3 (10)	1 (16.7)	3 (18.8)	11 (45.8)
Anticoagulation N, (%)	24 (82.8)	6 (100)	7 (43.8)	22 (91.7)
Thrombolysis or Endovascular Treatment N, (%)	2 (6.7)	0 (0)	3 (18.8)	7 (29.2)
Early outcome		NA		
*Stable n/N (%)*	2/21 (9.5)		0/10 (0)	2/20 (10)
*Improved n/N (%)*	18/21 (85.7)		7/10 (70)	17/20 (85)
*Death n/N (%)*	1/21 (4.8)		3/10 (30)	1/20 (5)
Late outcome				
*Normal n/N (%)*	10/14 (71.4)	0/2 (0)	2/5 (40)	4/13 (30.8)
*Stable n/N (%)*	3/14 (21.4)	1/2 (50)	2/5 (40)	6/13 (36.2)
*Improved n/N (%)*	1/14 (7.1)	0/0 (0)	1/5 (20)	3/13 (23.1)
*Death n/N (%)*	0/14 (0)	1/2 (50)	0/5 (0)	0/13 (0)

CNS: Central Nervous System, CVST: Cerebral Venous Sinus Thrombosis, SD: Standard Deviation.

## Data Availability

No data are available.
